# Liver Cirrhosis and Hepatocellular Carcinoma Diagnosed from Chylothorax: A Case Report

**DOI:** 10.3390/clinpract11030073

**Published:** 2021-09-03

**Authors:** Kenta Ito, Yoshimasa Hachisu, Mitsuhiko Shibasaki, Kazuma Ezawa, Hiroshi Iwashita, Asuka Jingu, Hirotaka Arai, Takeo Horie, Atsushi Takise

**Affiliations:** 1 Department of Respiratory Medicine, Maebashi Red Cross Hospital, 389-1, Asakura-Machi, Maebashi 371-0811, Japan; i10kenx2@gmail.com (K.I.); ema0317@outlook.jp (K.E.); h.iwa.shigoto@gmail.com (H.I.); tamaki.ja.schwarz@gmail.com (A.J.); t-horie@maebashi.jrc.or.jp (T.H.); at-takise@maebashi.jrc.or.jp (A.T.); 2 Department of Gastroenterology, Maebashi Red Cross Hospital, 389-1, Asakura-Machi, Maebashi 371-0811, Japan; shibao516@yahoo.co.jp (M.S.); harukaiyu0213@gmail.com (H.A.)

**Keywords:** chylothorax, liver cirrhosis, hepatocellular carcinoma, chylous ascites

## Abstract

A 71-year-old man visited our hospital with dyspnea and left pleural effusion. Left pleural effusion was diagnosed as chylothorax by thoracentesis. He had no history of trauma or surgery, and there were no findings of malignant lymphoma or thrombosis. Furthermore, he was diagnosed with liver cirrhosis and hepatocellular carcinoma by computed tomography and hematological examinations, and the chylothorax was considered to be caused by liver cirrhosis. We report a review of the literature with this case since it is relatively rare for cirrhosis and hepatocellular carcinoma diagnosed from chylothorax.

## 1. Introduction

There are many causes of pleural effusion, including infection, malignant tumors, heart failure, and trauma. Chylothorax is a condition caused by a disruption of the thoracic duct, resulting in leakage of chyle into the thoracic cavity [1]. It is characterized by a cloudy nature rich in triglycerides. Chylothorax is caused by trauma or surgery in many cases. However, in the absence of these physical causes, elucidation of the cause is essential.

Here, we report a case of cirrhosis and hepatocellular carcinoma diagnosed from chylothorax and review the relevant literature.

## 2. Case Presentation

**Figure 1:**
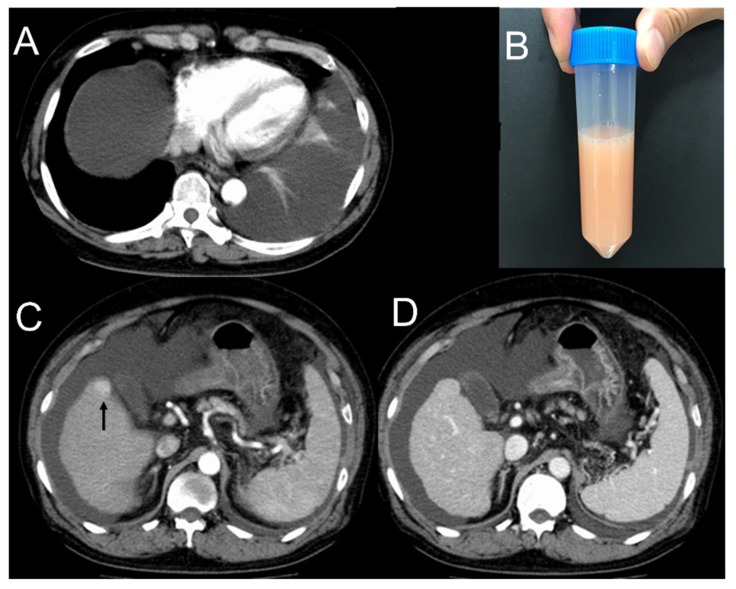
Imaging of chylothorax and computed tomography. (**A**) Computed tomography showed left pleural effusion and ascites. (**B**) The color of pleural effusion obtained by thoracentesis was milky white. (**C**,**D**) Contrast-enhanced computed tomography showed a 2-cm mass in the liver S6 that was enhanced in the arterial phase and washed out in the portal phase.

A 71-year-old man visited our hospital with a chief complaint of dyspnea for several days without other symptoms such as fever, cough, phlegm, or chest pain. He is a heavy drinker and had a history of diabetes mellitus and hypertension, but did not have a history of heart disease, respiratory disease, trauma, or surgery. Chest computed tomography (CT) showed a left pleural effusion and ascites (Figure 1A). There was no obvious pulmonary opacity suggestive of a tumor or infection. Serum white blood cell count or brain natriuretic hormone (BNP) were not increased. Laboratory findings did not show any evidence of infection or heart failure (Table 1). He was negative for hepatitis B virus antigen and hepatitis C virus antibody. Pleural effusion from thoracentesis was white and cloudy with high triglyceride levels, consistent with chylothorax (Figure 1B). The nature of pleural effusion was transudative, and cytology was negative for malignancy.

Abdominocentesis was performed, and milky-like ascites were considered to be chylous ascites. Lymphoscintigraphy did not reveal any apparent areas of leakage. Contrast-enhanced chest and abdominal CT revealed an irregular liver surface, consistent with cirrhosis, and a 2-cm-large mass in liver S6 with enhanced arterial phase, diagnosed with hepatocellular carcinoma (Figure 1C,D). There were no other findings suggestive of tumors or thrombosis in the portal vein. He was diagnosed with alcoholic cirrhosis because he was negative for the hepatitis virus and had a heavy alcohol history. The patient was diagnosed with chylothorax and chylous ascites due to liver cirrhosis.

**Figure 2:**
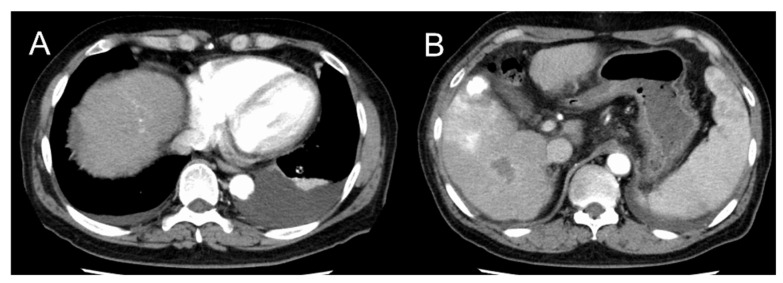
Imaging after two months of treatment for hepatocellular carcinoma. (**A**) Left pleural effusion and ascites have decreased compared to pretreatment. (**B**) There is lipiodol deposition coincident with the mass and no recurrence of hepatocellular carcinoma.

The pleural and ascitic effusion showed a marked reduction with the treatment of furosemide, spironolactone, and propranolol for two months without recurrence (Figure 2). The hepatocellular carcinoma, stage I, was treated with both radiofrequency ablation and transarterial chemoembolization, and the patient has been in remission.

## 3. Discussion

Chylothorax is a condition caused by a disruption of the thoracic duct and leakage of chyle into the thoracic cavity [1]. The non-traumatic causes of chylothorax include tumors and thrombosis, and malignant lymphoma is the most common cause of malignant disease [2]. Chylothorax due to malignant lymphoma is caused by the reflux of chyle due to the increased pressure in the thoracic duct. It can also be caused by a rupture of the thoracic duct due to vascular invasion of the lymphoma [3]. Liver cirrhosis, congestive heart failure, and nephrotic syndrome are also common causes of transudative chylothorax. In liver cirrhosis, increased portal pressure causes microscopic lymphatic destruction of intra-abdominal organs and the mesentery, resulting in chylous ascites. A mechanism has been proposed in which this fluid flows into the thoracic cavity through a diaphragmatic defect and appears as chylothorax [4]. Most cases of cirrhosis associated with chylothorax have been reported to be caused by viruses or alcohol, and there are few reports of nonalcoholic steato-hepatitis [4]. In a study including patients with cirrhosis-associated hepatic hydrothorax, 77% of the patients had only right-sided effusion, and 17% had only left-sided effusion [5]. The mechanism of the laterality of cirrhosis-associated chylothorax has not been well understood. However, all previously described cases of chylothorax have been reported as laterality of the right side [4,6,7]. This was similar to normal hepatic hydrothorax. The reason for the left-sided pleural effusion, in this case, cannot be clearly explained.

Treatment of non-traumatic chylothorax includes a low-fat diet, thoracic drainage, and thoracic duct embolization. Treatment of the primary disease is also essential in secondary chylothorax. Higuchi et al. reported that propranolol, a β-blocker, was administered to lower portal pressure in patients with cirrhosis. This resulted in an improvement of the chylothorax [8]. In patients with compensated cirrhosis and portal hypertension, it has been reported that lowering the portal-hepatic venous pressure gradient with β-blockers reduces the incidence of ascites. This prevents progression to non-compensated cirrhosis [9]. In our study, propranolol was administered to the patient in accordance with previous reports, and the chylothorax was reduced. Although other drugs may also be effective, β-blockers may effectively treat chylothorax associated with cirrhosis. It has been reported that transjugular intrahepatic portosystemic shunting was effective in the treatment of chylothorax associated with liver cirrhosis [10]. Tolvaptan has also been reported to be effective in the treatment of ascites associated with liver cirrhosis [11]. If his pleural and ascites effusions become more difficult to control, it may be considered as an additional treatment.

In this case, we could diagnose cirrhosis, child-pugh grade B, together with hepatocellular carcinoma as the cause of chylothorax. Romero et al. reported a case of chylothorax diagnosed with hepatocellular carcinoma two months before the patient died of the hepatorenal syndrome [12]. However, there are no reports of hepatocellular carcinoma diagnosed on occasion with chylothorax. Chronic lymphocytic leukemia, primary lung carcinoma, and metastatic lung carcinoma are also known as the causes of chylothorax [2]. However, the coexistence of chylothorax with intra-abdominal parenchymal tumors has not been well known. When hepatocellular carcinoma invades directly into the portal vein, it is considered that the hepatocellular carcinoma itself may cause portal hypertension. However, no such finding was observed in this case. It is not clear to what extent hepatocellular carcinoma affected chylothorax associated with cirrhosis. Although liver cirrhosis rather than hepatocellular carcinoma may be more likely associated with chylothorax in our case, the presence of hepatocellular carcinoma in the case of cirrhosis with chylothorax should be considered.

## 4. Conclusions

When we diagnose transudative chylothorax, we should consider the complication of liver cirrhosis. In addition, the possibility of the presence of hepatocellular carcinoma should be kept in mind.

## Figures and Tables

**Table 1:** Laboratory data at the first visit.

Hematology			Biochemistry			Plural Effusion		
White blood cells	2700	/μL	Total protein	5.8	g/dL	Color	Milky white	
Neutrophils	70.3	%	Albumin	2.9	g/dL	pH	8.5	
Lymphocytes	13.3	%	Total bilirubin	1.1	mg/dL	White blood cells	500	/μL
Monocytes	12.3	%	Direct bilirubin	0.1	mg/dL	Neutrophils	5.0	%
Eosinophils	3.7	%	AST	38	U/L	Lymphocytes	60.0	%
Basophils	0.4	%	ALT	33	U/L	Monocytes	33.0	%
Red blood cells	346 × 10^4^	/μL	LDH	213	U/L	Eosinophils	2.0	%
Hemoglobin	8.7	g/dL	ALP	121	U/L	Hematocrit	0.1	%
Hematocrit	28.2	%	γ-GTP	42	U/L	Total protein	1.6	g/dL
Platelets	30.4 × 10^4^	/μL	CK	153	U/L	LDH	66	U/L
			BUN	11	mg/dL	Glucose	189	mg/dL
Coagulation			Creatinine	0.74	mg/dL	Total cholesterol	30	mg/dL
PT%	64	%	Sodium	139	mEq/L	Triglyceride	227	mg/dL
PT-INR	1.25		Potassium	3.6	mEq/L	ADA	9	U/L
APTT	28.9	sec	Chloride	107	mEq/L			
Fibrinogen	225	mg/dL	Calcium	7.8	mg/dL	Cytology		(−)
D-dimer	13.3	μg/mL	Glucose	198	mg/dL			
			HbA1c	5.9	%	Culture		
Tumor marker			Total cholesterol	99	mg/dL	Routine		(−)
AFP	69.7	ng/mL	LDL-cholesterol	70	mg/dL	Mycobacterium		(−)
CEA	1.5	ng/mL	HDL-cholesterol	26	mg/dL			
CA19-9	36.9	U/mL	Triglyceride	56	mg/dL			
Soluble IL-2 receptor	842	U/mL	BNP	34.4	pg/mL			
			CRP	1.46	mg/dL			
Virus marker			ANA	40	titer			
HBV antigen	(−)							
HCV antibody	(−)							

PT: Prothrombin time, APTT: Activated partial thromboplastin time, AFP: α-fetoprotein, CEA: Carcinoembryonic antigen, CA19-9: Carbohydrate antigen 19-9, HBV: Hepatitis B virus, HCV: Hepatitis C virus, AST: Aspartate aminotransferase, ALT: Alanine aminotransferase, LDH: Lactate dehydrogenase, ALP: Alkaline phosphatase, γ-GTP: γ-glutamyl transpeptidase, CK: Creatine kinase, BUN: Blood urea nitrogen, HbA1c: Hemoglobin A1c, LDL: Low-density lipoprotein, HDL: High-density lipoprotein, BNP: Brain natriuretic peptide, CRP: C-reactive protein, ANA: Anti-nuclear antibody, ADA: Adenosine deaminase.
